# H.264 SVC Complexity Reduction Based on Likelihood Mode Decision

**DOI:** 10.1155/2015/418437

**Published:** 2015-06-28

**Authors:** L. Balaji, K. K. Thyagharajan

**Affiliations:** ^1^Faculty of Information & Communication, Anna University, Chennai 600025, India; ^2^Department of ECE, Velammal Institute of Technology, Panchetti, Tamil Nadu 601204, India; ^3^RMD Engineering College, Kavaraipettai, Tamil Nadu 601206, India

## Abstract

H.264 Advanced Video Coding (AVC) was prolonged to Scalable Video Coding (SVC). SVC executes in different electronics gadgets such as personal computer, HDTV, SDTV, IPTV, and full-HDTV in which user demands various scaling of the same content. The various scaling is resolution, frame rate, quality, heterogeneous networks, bandwidth, and so forth. Scaling consumes more encoding time and computational complexity during mode selection. In this paper, to reduce encoding time and computational complexity, a fast mode decision algorithm based on likelihood mode decision (LMD) is proposed. LMD is evaluated in both temporal and spatial scaling. From the results, we conclude that LMD performs well, when compared to the previous fast mode decision algorithms. The comparison parameters are time, PSNR, and bit rate. LMD achieve time saving of 66.65% with 0.05% detriment in PSNR and 0.17% increment in bit rate compared with the full search method.

## 1. Introduction

H.264 Scalable Video Coding (SVC) as an elongation of H.264 Advanced Video Coding (AVC) permits a single encoding but multiple decoding capabilities [1] of various gadget requirements. SVC prolongs all the characteristics of AVC; in addition to that, it provides a multiple layered approach, efficiency in coding, and so forth. The multiple layered approach constitutes base layer and one or more enhancement layers. The base layer consists of more essential information in the form of bit stream. The bit stream is partitioned off into more amounts of subset bit streams [1] known as enhancement layer. The subset bit stream comprises only essential message of the video while removing all redundant and less essential messages. The less essential message is deduced from base layer and already coded enhancement layers [1]. The base layer contains a bit stream of low resolution or low frame rate or low quality. The enhanced resolution or frame rate or quality will be obtained by adding enhancement layer bit streams.

SVC can be able to decode the video content, even with a limited bit stream of its exclusive feature, referred to as scalability. Scalability in SVC undergoes three levels: spatial, temporal, and quality. Spatial scalability refers to the resolution or the dimension of the video. Temporal scalability refers to the number of frames per second in the video. Quality or SNR scalability refers to PSNR (peak signal to noise ratio) gain in the video. SVC executes temporally with hierarchical B picture prediction of frames. A frame in a video is categorized into macroblocks (MBs). A macroblock contains many blocks of modes in which each has its own identity. The temporal scalability performs mode search for a prime mode in a macroblock (MB). SVC constitutes three types of frames, such as I or intraframe, P or prediction frame, and B or bidirectional prediction frames. I frame constitutes more essential information which requires all the modes in a macroblock to be coded. P frames contain essential information, but less compared to I frame, which requires few modes to be coded. B frames contain less essential information which requires very few modes to be coded.

The frames are divided in terms of fixed size macroblocks of 16 × 16 in the former standard. But, in H.264/AVC, variable block sizes of 16 × 16, 16 × 8, 8 × 16, 8 × 8, 8 × 4, 4 × 8, and 4 × 4 are available. It also offers to have its own way of estimating the modes on how the macroblock is divided. The prime mode for a MB or block will be decided based on rate distortion cost (RDC) function using Lagrangian parameter. RDC computation includes integer transform, quantization, and entropy coding in both forward and backward process. RDC computed for all the modes in a macroblock and the mode with minimum value is decided as the prime mode in a MB or block. SVC defines nine intramodes for prediction in a block of INTRA 4 × 4, four intramodes for prediction in a macroblock of INTRA 16 × 16, seven intermodes for prediction in a macroblock of INTER 16 × 16, INTER 16 × 8, INTER 8 × 16, INTER 8 × 8, INTER 8 × 4, INTER 4 × 8, and INTER 4 × 4, and one SKIP mode [2]. For motion estimation, BL_PRED mode and QPEL_REF mode for enhancement layer are added to the modes of the base layer. A full search method decides upon the ideal motion vector difference (MVD) using RDC between the current frame and the previous frame. The MVD is the difference between a predicted motion vector and actual motion vector between current and previous frame. The computationally in-depth rate distortion optimization method increases encoding time and complexity and results in many fast motion decision algorithms to develop. More algorithms are evaluated for AVC which is less difficult compared to SVC. But few algorithms are evaluated for SVC which saves time while selecting a prime mode. These algorithms are discussed in the next section.

## 2. Related Work

The complexity in determining the prime mode in H.264/AVC is proposed [3], which saves encoding time. The proposed method involves Lagrangian optimization with rate and distortion cost to decide the prime mode while achieving less encoding time and coding efficiency. A motion activity-based mode decision (MAMD) algorithm is proposed in [4] to speed up the coding time by minimizing the number of candidate modes. The candidate modes are skipped based on motion vectors, avoiding them to be coded, thus reducing time. The candidate modes of the enhancement layer are significantly lessened on the relation between the base layer and the enhancement layer in [5]. But, base layer modes are chosen based on full search process. A probability based coding mode decision algorithm [6] is accomplished for H.264/AVC. The mode is resolved with the maximum probability of correlation between the adjacent block and present block. The probability model saves more encoding time. A timely outcome of mode decision is proposed for the enhancement layer MB in [7]. If the MB is found to be all zeros, then the previous MB can be chosen and the mode decision method can be earlier terminated.

The enhancement layer MB mode is determined using the Bayesian theorem, proposed in [8]. The proposed algorithm discusses the Markov procedure. The Markov procedure based likelihood analysis finds the mode for a macroblock earlier and saves time. The correlation among adjacent MBs of the base layer and the colocated MBs of enhancement layer is utilized to forecast the mode in the enhancement layer in [9]. A selective interlayer residual prediction using Lagrangian RDC based fast mode decision algorithm is proposed in [10]. The Lagrangian parameter involved in this prediction reduces coding time while deciding appropriate mode. Classification based intra-inter mode decision is accomplished in [11]. The frame will be coded or skipped based on the determination of intra-inter coding for rate control. This approach is devoted to video over networks and then bestowed to scalable video coding. In [12], the relation between MB of enhancement layer and its colocated base layer MB is used for mode decision. Intermode prediction for temporal scalability is proposed in [13]. The proposed method compares the pixel values of the current MB with reference block using statistical analysis. In our previous works [14], a desired mode list is constructed for predicting the mode in the base layer. The mode for enhancement layer is predicted based on correlation between current frame and reference frame. A quick video streaming through the Internet is accomplished using a mathematical model in [15]. The mathematical model maximizes the information rate to the client from the streaming server, which plays a delay-free video.

Although each proposed algorithm evaluates faster encoding time in deciding the prime mode, it fails to fulfill in terms of PSNR and bit rate with the full search method. Only a comparative measure was obtained among different algorithms in terms of encoding time, irrespective of the computational complexity involved. As a result, a fast mode decision algorithm with low computation complexity which attains less encoding time is proposed. The proposed algorithm uses likelihood mode decision method, discussed in the next section.

## 3. Likelihood Mode Decision

In SVC, the rate distortion cost (RDC) based mode decision is performed. The mode with minimum RDC will be decided as prime mode for each MB in full search method. But the complexity in estimating RDC for each mode in a MB is tedious, in turn consuming more encoding time. To decide a prime mode earlier and escape from encoding unwanted modes are the question to be discussed. In this section, a likelihood mode decision (LMD) algorithm is proposed which decides the prime mode for I frame of the enhancement layer. P/B frames which are derived from I frames need less attention. Also, these frames hold less essential information; a selective prediction of modes can be implemented for obtaining the prime mode. The I frame of base layer is of more importance which follows a standard full search algorithm, while P/B frames involve an enhanced selective prediction of certain modes.

The likelihood model is evaluated below to show the importance of likeliness in terms of intermode prediction. The likeliness of modes between adjacent and current MB resembles high degree of likeliness to be same mode. The video sequence tested with various quantization parameters for different MB is disclosed in Table 1.

Let MB(*j*, *x*, *y*) represent a MB available at the top left pixel of *j*th frame. MBs will be available from (0, 0) to (±16, ± 16) in (*x*, *y*) around a current block as shown in Table 1. These MBs will have a similar degree of likeliness with current *j*th frame, previous (*j* − 1)th frame, and previous colocated MB (*j* − 1′)th frame with current coding MB. Each MB around current coding MB will have its average likeliness of the same mode. From the observation, the intermodes tend to have same mode resemblance with minimum of five MBs in order. The chosen five MBs in order will decide the prime mode for a MB. So, we define a MB set *P* with these five MBs:

(1) P=MBj,x−16,y−16,MBj,x−16,y,MBj,x,y−16,MBj,x+16,y−16,MBj−1′,x,y,

where MB(*j*, *x*, *y*) represents the MB located on the *j*th frame with the upper left pixel at *x*, *y* and MB((*j* − 1)′, *x*, *y*) represents the former colocated MB with same as current coding MB. We define adjacent mode set *Q* by

(2) $$\begin{array}{c} Q=\left\{\;M_{MB}\;|\;MB\in P\;\right\}, \end{array}$$

where *M*_MB_ represents the encoding mode of MB. The proximate likelihood of the mode to be a prime mode is assumed by the likelihood model:

(3) Rm=M ∣ M∈Q≈Rm∈Q∑MB∈P,MMB=mNm=MMB∑m∈QMB∈P∑MMB=mNm=MMB=K·∑MB∈P,MMB=MNm=MMB,

where *N*(·) is occurrence measure of an event and *K* is constant argument which is same for all modes. Since *M* is not a member of *Q*, the state may have less likelihood to be prime mode and is observed to be nothing. With the likelihood standard explained in declaration (3), a few numbers of intermodes present in set *Q* have more likelihood to be prime mode.

**Figure 1:**
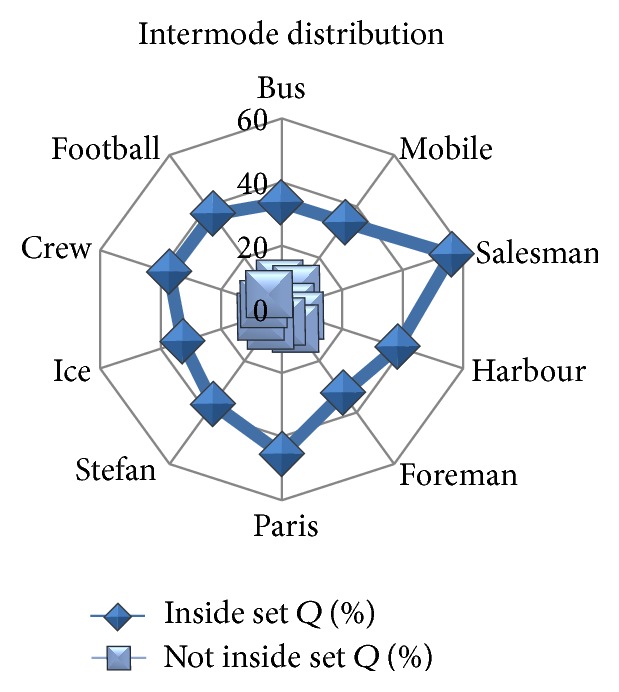
Intermode distribution over average likeliness.

Table 2 discloses the outcome of the likelihood standard when addressed for the video sequences. The set of intermodes in set *Q* has the full likeliness of being prime mode. The intermodes not in set *Q* will have less likelihood to be prime mode. A contrastive analysis of standard likelihood with the video sequences of intermode distribution is shown in Figure 1.

Video sequences with both fast and slow motion have a high percent distribution of likeliness to be the prime mode in set *Q*. From the experimental observation, we conclude to search in set *Q* for prime mode in the beginning. This will significantly reduce the encoding time compared with the full search method. Also the video sequence which has slow motion has a higher likeliness than with fast motion. This indicates that the likelihood model works well with sequences having less motion vector differences.

### 3.1. Implementation of LMD Algorithm

**Figure 2:**
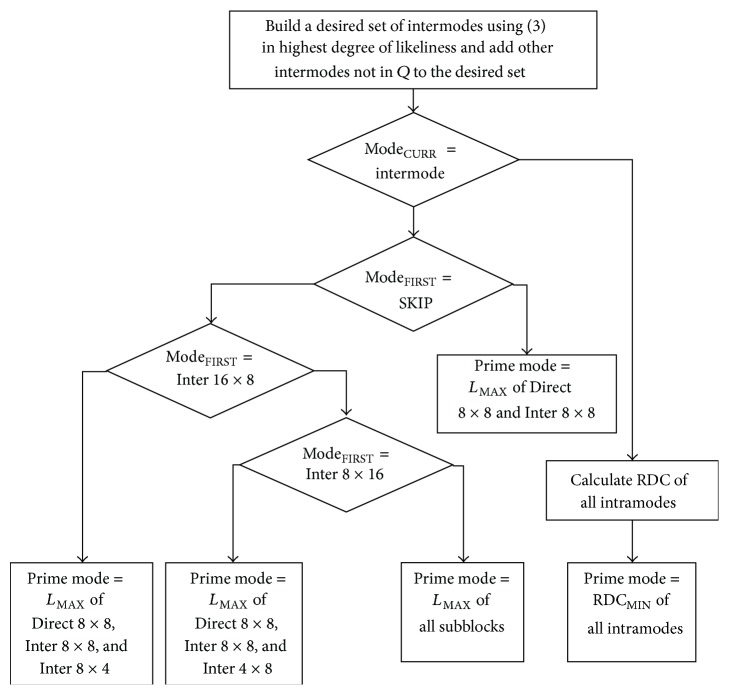
Flowchart of likelihood mode decision algorithm.

A desired set of intermodes with maximum likeliness is built using (3) from set *Q*. The intermodes in the set are sorted in the order of the highest degree of likeliness. The intermodes not in set *Q* are added up in the order of SKIP, Inter 16 × 16, Inter 16 × 8, Inter 8 × 16, and subblocks to the desired set. The mode with maximum likeliness will be a prime mode. The LMD algorithm applied for I or intraframe of enhancement layer which involves more interlayer predictions. This likelihood model will further reduce the encoding time and achieve early termination.  Figure 2 shows the flowchart of LMD algorithm. The proposed algorithm initially checks for intermode; having checked, it decides upon the first mode of a macroblock. The first mode of a macroblock is SKIP; then there is no need to encode the mode; if Inter 16 × 8, the prime mode will be maximum likeliness of Direct 8 × 8, Inter 8 × 8, and Inter 8 × 4; if Inter 8 × 16, the prime mode will be maximum likeliness of Direct 8 × 8, Inter 8 × 8, and Inter 4 × 8; else check for all subblocks. The prime mode for intramodes will be computed based on minimum RDC.

**Figure 3:**
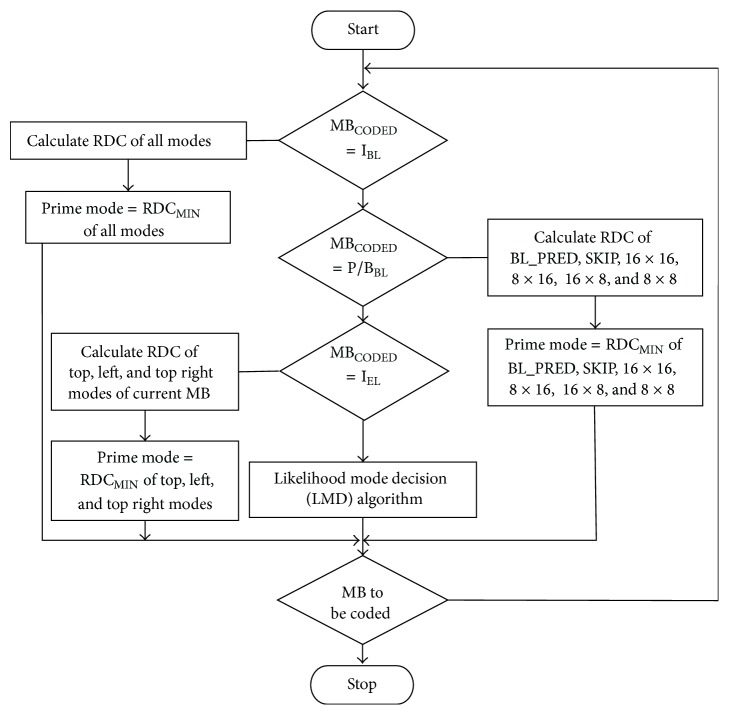
Flowchart of fast mode decision algorithm for SVC.

A selective prediction is accomplished for P/B frames of the enhancement layer. Since P/B frames have less information than I frame, these frames can be coded in a less efficient manner.  Figure 3 shows the proposed flowchart of the fast mode decision algorithm with LMD. The top, left, and top right modes of a macroblock RDC are computed. The prime mode will be chosen with a minimum RDC among these modes. Meanwhile, a standard search algorithm is implemented for base layer. If the encoding MB was found to be I frame in base layer, mode decision will be minimum RDC of all modes, while P/B frames mode decisions include minimum RDC of BL_PRED, SKIP, 16 × 16, 16 × 8, 8 × 16, and 8 × 8 modes.

The algorithm describes Likelihood Mode Decision for I frames and selective prediction for P/B frames of enhancement layer, whereas a standard full search method is implemented for I frames and an enhanced selective prediction for P/B frames of base layer.

### 3.2. Pseudocode

See Pseudocode 1.

## 4. Experimental Results

The proposed fast mode decision algorithm was evaluated using JSVM reference software 9.19.15 [16] with the simulation parameters as in Table 3. The system configuration uses Intel Core i3 Processor with 2.4 Ghz clock speed and 500 GB hard disk with Windows 7 operating system. We use the following Bus, City, Crew, Football, Foreman, Harbour, Mobile, and Soccer video sequences for the proposed algorithm. All video sequences were implemented both spatially and temporally. QCIF (Quadrature Common Intermediate Format) is for base layer with 15 frames per second and CIF (Common Intermediate Format) is for enhancement layer with 30 frames per second. The combined spatial and temporal implementation of the proposed algorithm has the different sets of quantization parameters. The quantization parameters ranging between 18/22, 28/32, and 38/42 for base and enhancement layer with GOP size set to 16. The search range for a mode in a macroblock is 32.

The performance of each video sequence is measured based on the average encoding time (ΔTime) in seconds using (4), average luminance peak signal to noise ratio (ΔYPSNR) in dB using (5), and average bit rate (ΔBit rate) in kbps using (6) as stated below:

(4) ΔTime=Timeproposed−TimeoriginalTimeoriginal∗100,

(5) ΔYPSNR=YPSNRproposed−YPSNRoriginal,

(6) ΔBit  rate=Bit  rateproposed−Bit  rateoriginalBit  rateoriginal∗100.

The experimental result for each video sequence under different quantization parameters is shown in Table 4. Each video sequence is evaluated under three measures such as ΔYPSNR, ΔBit  rate, and ΔTime. The measures are obtained for the proposed algorithm and compared with the previous algorithms as shown in Table 4.

**Figure 4:**
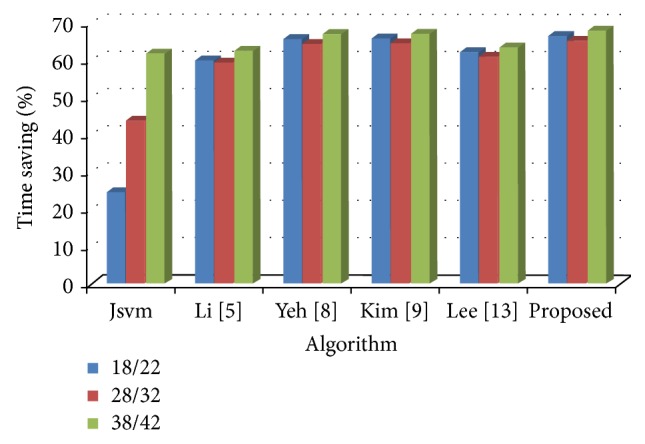
Time saving relationship among different algorithms for various values of QP.

From the observation, the proposed algorithm achieves better encoding time of 66.65% with 0.05% detriment in PSNR and 0.17% increment in bit rate compared with the full search method. This measure is an average value achieved among various quantization parameters chosen in the experiment. It also outperforms all other previous fast mode decision algorithms in terms of encoding time and few algorithms in addition with PSNR and bit rate.  Figure 4 shows the percentage time saving relations between video sequences among the proposed and previous algorithms. All algorithms, including the full search method, save encoding time when the value of quantization parameter is high for base and enhancement layer. The full search method encoding time will be more, when the value of quantization parameter is less. In Figure 4, the algorithm shows the amount of time saving obtained even at a low quantization parameter. On an average, the proposed algorithm saves maximum time saving compared to the previous works.

**Figure 5:**
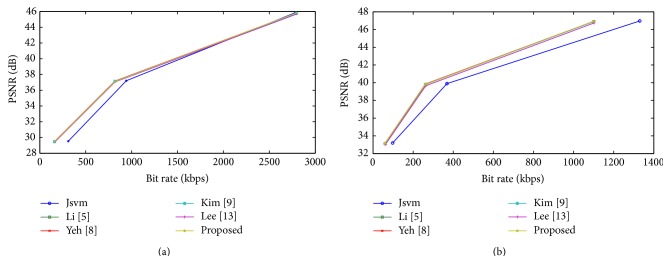
Rate distortion curves for (a) Bus and (b) Crew.

**Figure 6:**
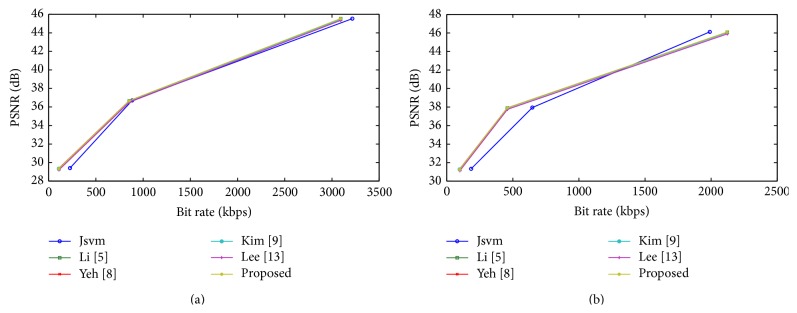
Rate distortion curves for (a) Harbour and (b) Soccer.

**Figure 7:**
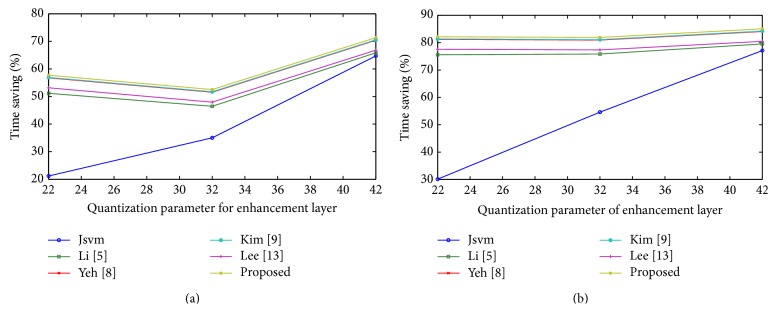
Time saving curves for (a) Football and (b) Foreman.

**Figure 8:**
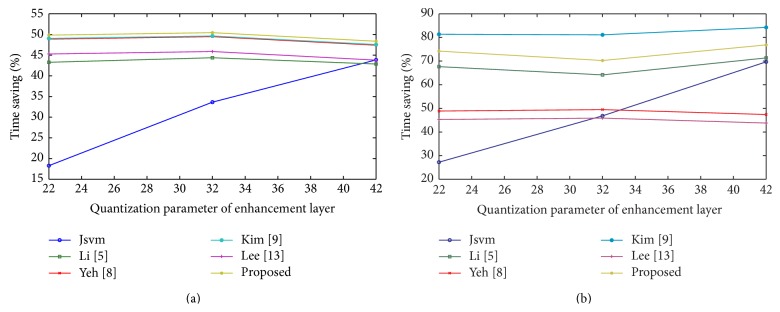
Time saving curves for (a) Mobile and (b) Soccer.

The rate distortion curves are shown in Figures 5 and 6 for Bus and Crew; Harbour and Soccer sequence, respectively. For all sequences, among all FMD algorithms, a small deterioration in PSNR and bit rate is suffered, compared with the full search algorithm. Although, the proposed algorithm deteriorates in PSNR and bit rate, it achieves an acceptable level of quality with other fast mode decision algorithms. Figures 7 and 8 show the time saving curves for Football and Foreman; Mobile and Soccer sequence, respectively. From the observation, under each sequence, the full search method encoding time is more for low QP, with less time for high QP. Even the full search method achieves minimum encoding time under high QP; it is lesser when compared to other FMD algorithms. But, in general, all FMD algorithms outperform full search method at low QP itself. The proposed algorithm achieves maximum time saving for almost all video sequences. Kim's algorithm achieves faster encoding time for Soccer sequence next to the proposed algorithm.

Here, three cases are to be compared with the values of quantization parameter for the base and enhancement layer: case 1: the sequence with gradual increase in time saving as QP increases; case 2: the sequence with gradual decrease in time saving as QP increases; and case 3: gradual difference in time saving as QP increases. In case 1, the Harbour sequence falls under this category which is a slow motion sequence with less number of MVDs; in case 2, the city sequence is also a slow motion sequence, but with more number of MVDs; and, in case 3, all other sequences such as Bus, Crew, Football, Foreman, Mobile, and Soccer have an average number of MVDs. Even though Foreman, a fast motion sequence, achieves maximum time saving, it is due to the average amount of MVDs; while Mobile, a slow motion sequence, achieves minimum time saving with the same average amount of MVDs and it is due to large MVDs.

Under case 1, it involves less number of I frames and number of P/B frames in the enhancement layer, which involves likelihood model for few I frames, while, under case 2, it involves number of I frames and less number of P/B frames, which involves likelihood model for more I frames. In case 3, irrespective of the sequence, fast or slow, it achieves maximum and minimum time saving based on MVDs globally.

Hence we conclude that the proposed algorithm can achieve maximum time saving for fast motion sequence with lesser amount of MVDs and an average time saving can be realized for slow motion sequence with larger amount of MVDs. Meanwhile, all other sequences realized to produce maximum encoding time. The PSNR deteriorates a bit lower with an acceptable signal level and an increase in bit rate compared to the full search algorithm. It can be compromised with the gain of abundant saving in encoding time.

## 5. Conclusion

The experimental observation depicts that the proposed algorithm achieves faster encoding time, for both fast and slow motion video sequence. The evaluated measures are compared with the full search method and previous FMD algorithms. The comparison shows that the proposed algorithm outperforms the full search method and other previous algorithms. A desired set of intermodes, built for I frame in the enhancement layer, reduces the computation complexity, thus decreasing encoding time, whereas P/B frames of enhancement layer, with less information, escape from the exhaustive full search method by selective prediction, further reducing encoding time. The future enhancement of the work will enforce achieving same PSNR and bit rate of the full search method with faster encoding time.

## Figures and Tables

**Pseudocode 1:**
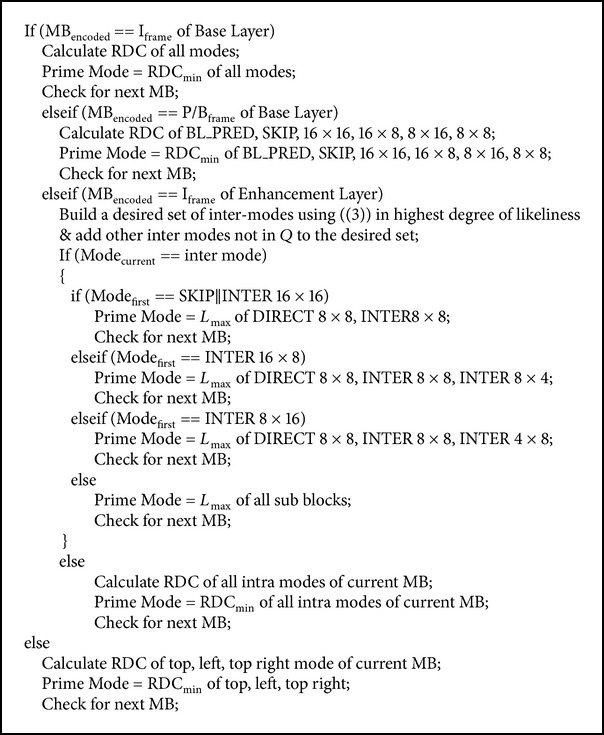


**Table 1:** Macroblock average likeliness under various QP.

Macroblock in *j*th frame	Average likeliness (%)			
	QP = 30	QP = 34	QP = 38	QP = 42
MB(*j*, *x* − 16, *y* − 16)	41.1	47.8	61	69.4
MB(*j*, *x* − 16, *y*)	49.7	56.8	68.3	76.8
MB(*j*, *x*, *y* − 16)	43.1	50.5	63.4	72.1
MB(*j*, *x* + 16, *y* − 16)	40.5	48.2	61	70.1
MB(*j* − 1′, *x*, *y*)	51.5	59.3	72.7	80.1
MB(*j* − 1, *x*, *y*)	35.9	35.5	38.2	39.4
MB(*j* − 1, *x* − 16, *y* − 16)	28.7	28.9	31.9	33.6
MB(*j* − 1, *x* − 16, *y*)	32.2	31.6	34.8	37
MB(*j* − 1, *x* − 16, *y* + 16)	29.4	28.8	32	33.9
MB(*j* − 1, *x*, *y* − 16)	29.9	30	32.9	34.9
MB(*j* − 1, *x*, *y* + 16)	30.2	29.9	32.9	34.8
MB(*j* − 1, *x* + 16, *y* − 16)	28.8	29.5	32	34.4
MB(*j* − 1, *x* + 16, *y*)	28.7	31.3	34.6	36.9
MB(*j* − 1, *x* + 16, *y* + 16)	28.9	28.7	32.2	34

**Table 2:** Intermode distribution based on average likeliness.

Video sequence	Mode distribution over likeliness	
	Intermodes in set *Q* (%)	Intermodes not in set *Q* (%)
Bus	33.65	8.08
Mobile	33.86	7.92
Salesman	56.12	2.45
Harbour	38.21	6.39
Foreman	32.66	7.41
Paris	45.48	3.82
Stefan	36.74	6.2
Ice	32.87	7.19
Crew	37.23	5.48
Football	36.88	6.38

**Table 3:** Simulation parameters.

Simulation conditions	
Resolution	
Base layer	QCIF
Enhancement layer	CIF

Frame rate	
Base layer	15 Hz
Enhancement layer	30 Hz

Encoding options	Search range: 32
	Number of frames: 150
	Reference frame number: 1
	GOP size: 16

Encoder/decoder	JSVM 9.19.15

**Table 4:** Simulation results of combined spatial and temporal scalability of various algorithms.

Sequence	QP	ΔYPSNR (dB)					ΔBit rate (kbps)					ΔTime (%)				
	BL/EL	Li [5]	Yeh [8]	Kim [9]	Lee [13]	Prop.	Li [5]	Yeh [8]	Kim [9]	Lee [13]	Prop.	Li [5]	Yeh [8]	Kim [9]	Lee [13]	Prop.
Bus	18/22	−0.004	−0.046	−0.092	−0.183	−0.028	1.665	0.797	0.510	2.802	0.788	65.56	56.76	68.54	63.67	72.12
	28/32	−0.003	−0.037	−0.074	−0.149	−0.109	1.072	0.205	−0.053	2.209	0.196	65.85	51.56	70.33	48.32	71.91
	38/42	−0.002	−0.030	−0.059	−0.118	−0.060	0.934	−0.529	−0.041	1.475	−0.538	71.02	70.41	61.98	41.23	76.53

City	18/22	−0.005	−0.046	−0.092	−0.184	−0.030	2.019	0.411	0.779	2.322	0.309	61.67	73.24	67.43	67.56	68.23
	28/32	−0.003	−0.038	−0.076	−0.153	−0.071	1.008	0.135	0.187	1.938	−0.076	46.82	69.27	52.08	67.35	52.88
	38/42	−0.003	−0.031	−0.062	−0.124	−0.085	0.750	−1.666	−0.547	0.889	−1.124	40.28	75.88	44.99	71.97	45.79

Crew	18/22	−0.005	−0.047	−0.094	−0.188	−0.048	3.826	0.528	0.664	3.963	1.950	62.78	81.18	56.92	55.19	69.34
	28/32	−0.004	−0.040	−0.080	−0.160	−0.082	3.409	−0.035	0.486	3.545	1.532	65.07	80.94	51.72	67.09	71.13
	38/42	−0.003	−0.033	−0.066	−0.133	−0.051	2.761	−0.023	−0.410	2.898	0.885	57.28	84.07	70.57	72.39	62.78

Football	18/22	−0.006	−0.046	−0.092	−0.183	−0.001	2.549	1.959	0.425	2.686	0.673	51.16	67.27	73.40	53.16	57.72
	28/32	−0.005	−0.037	−0.073	−0.146	−0.048	2.372	1.541	0.459	2.509	0.495	46.46	51.92	69.43	47.96	52.52
	38/42	−0.003	−0.029	−0.057	−0.114	−0.073	1.475	0.894	−0.564	1.612	−0.401	65.86	44.83	76.04	66.81	71.37

Foreman	18/22	−0.004	−0.047	−0.093	−0.186	−0.085	2.279	0.682	0.393	2.416	0.402	75.58	68.38	71.32	64.78	82.14
	28/32	−0.003	−0.039	−0.078	−0.156	−0.040	2.002	0.504	0.117	2.139	0.126	75.84	70.17	71.11	66.57	81.90
	38/42	−0.003	−0.032	−0.065	−0.129	−0.123	0.202	−0.392	−1.684	0.338	−1.675	79.53	61.82	75.73	58.22	85.03

Harbour	18/22	−0.006	−0.046	−0.091	−0.182	−0.003	2.417	0.449	0.431	2.453	0.440	53.19	58.79	58.95	69.64	59.75
	28/32	−0.005	−0.037	−0.073	−0.147	−0.046	2.006	0.189	0.171	2.193	0.180	65.59	70.69	70.85	65.67	71.65
	38/42	−0.004	−0.029	−0.059	−0.118	−0.073	1.201	−0.653	−0.671	1.351	−0.662	71.45	75.99	76.15	72.28	76.95

Mobile	18/22	0.000	−0.046	−0.091	−0.182	0.001	1.395	0.318	0.300	2.532	0.519	43.27	71.16	49.03	77.58	49.83
	28/32	0.000	−0.037	−0.073	−0.147	−0.027	0.832	−0.067	−0.085	1.969	−0.044	44.37	70.95	49.63	77.34	50.43
	38/42	0.000	−0.029	−0.058	−0.116	−0.091	0.084	−1.115	−1.133	1.981	−0.032	42.85	75.57	47.55	80.47	48.35

Soccer	18/22	−0.004	−0.046	−0.092	−0.184	−0.032	4.311	0.443	1.941	2.448	0.434	67.64	48.87	81.34	45.27	74.20
	28/32	−0.004	−0.038	−0.076	−0.152	−0.041	3.344	0.477	1.523	2.481	0.468	64.17	49.47	81.10	45.87	70.23
	38/42	−0.003	−0.031	−0.063	−0.125	−0.079	2.321	−0.546	0.876	1.458	−0.555	71.33	47.39	84.23	43.79	76.84

Average	18/22	**−0.004**	**−0.046**	**−0.092**	**−0.184**	**−0.028**	**2.558**	**0.698**	**0.680**	**2.703**	**0.689**	**60.10**	**65.70**	**65.86**	**62.10**	**66.67**
	28/32	**−0.003**	**−0.038**	**−0.076**	**−0.151**	**−0.058**	**2.006**	**0.369**	**0.351**	**2.373**	**0.360**	**59.27**	**64.37**	**64.53**	**60.77**	**65.33**
	38/42	**−0.003**	**−0.031**	**−0.061**	**−0.122**	**−0.079**	**1.216**	**−0.504**	**−0.522**	**1.500**	**−0.513**	**62.45**	**67.00**	**67.16**	**63.40**	**67.96**
